# Cytotoxic Effect of Iranian *Vipera lebetina* Snake Venom on HUVEC Cells 

**Published:** 2015

**Authors:** Maryam Kakanj, Mahmoud Ghazi-Khansari, Abbas Zare Mirakabadi, Bahram Daraei, Hossein Vatanpour

**Affiliations:** a *Toxicology and Pharmacology Department, School of Pharmacy, Medical Science University of Shahid Beheshti, Tehran, Iran. *; b *Pharmacology Department, School of Medicine, Tehran University of Medical Sciences, Tehran, Iran. *; c *Venomous Animals and Antivenom Production Department, Razi Vaccine and Serum Research Institute, Karaj, Iran. *; d *Toxicology Department, Faculty of Medical Sciences, Tarbiat Modares University, Tehran, Iran.*

**Keywords:** Snake venom, Cytotoxicity effect, HUVEC, Hemorrhage, *V. lebetina*

## Abstract

Objective: Envenomation by heamotoxic snakes constituted a critical health occurrence in the world. Bleeding is the most sever consequence following snake bite with viperid and crothalid snakes. It is believed that the degradation of vascular membrane caused hemorrhage; in contrast, some suggested that direct cytotoxicity has role in endothelial cell disturbances. This study was carried out to evaluate the direct toxicity effect of *V. lebetina* crude venom on Human Umbilical Vein Endothelial Cells (HUVECs). Methods: The effect of *V. lebetina* snake venom on HUVECs growth inhibition was determined by MTT assay and neutral red uptake assay. The integrity of cell membrane through LDH release was measured with the Cytotoxicity Detection Kit. Morphological changes of endothelial cells were also evaluated using a phase contrast microscope. Result: In MTT assay, crude venom showed a cytotoxic effect on endothelial cells which was confirmed by the effect observed with neutral red assay. Also, crude venom caused changes in the integrity of cell membrane by LDH release. The morphological alterations enhanced in high concentration results in total cells number reduced. Conclusion: *V. lebetina* venom showed potential direct cytotoxic effects on human endothelial cells in a manner of concentration- dependent inhibition.

## Introduction

Snake bite is a major public health hazard in rural regions of tropical and subtropical areas in Africa, Asia and Latin America. It has been pointed out that at least 5.5 million snake bites lead to 125,000 deaths yearly around the world (1). According to the World Health Organization (WHO) in 2010, there are about 3000 species of snakes in the world and about 600 of which are classified as "venomous" that are potentially fatal. Over 200 are considered to be medically important (2). Among venomous snakes, the most important groups causing envenomation are Elapidae, Crotalidae and Viperidae (3). There are 25 out of 83 snake species in Iran which are venomous. There were about 53,787 snake envenomations that caused 67 mortalities in Iran within 2002-20011 (4). Due to pharmacological and physiological effects of snake venom, recently they are considered potentially as the pharmacological tools or agents affecting on different mammalian organs and human cell cultures in different research and development laboratories. Snake venoms are concentrated in specific venomous glands and they consist of different compounds, including proteins and peptides that have pharmacological and physiological diverse roles in mammalian body (5, 6). Some Toxins affecting the hematopoietic system, haemotoxins, isolated from snake venom. These toxins can cause bleeding with various effects at the site of bites lead to hemorrhage in organs such as heart, lungs, kidneys and brain (7, 8, and 9). Venoms from Viperidae and Crotalidae affect the human haemostatic system or disturb the endothelium cells. Hydrolysis of micro-vessel and homeostasis interruptions caused severe bleeding (10, 11). There are several models to study of cytotoxicity mechanisms caused by snake venom which is important to explain the venom pathophysiology (12). In this study we have examined the cytotoxicity of *Vipera Lebetina* crude venom on human umbilical vein endothelial cells.

## Experimental

*Venom and chemicals*

Iranian *V. lebetina* was obtained from Venomous Animals and Antivenin Production Dep., Razi institute of vaccine research and serum production, Karaj- Iran. Human Umbilical Vein Endothelial Cell line (HUVEC) was purchased from the National Cell Bank, Pasteur Institute of Iran. 3-(4, 5-dimethylthiaol-2-yl)-2, 5-diphenyltetrazolium bromide (MTT), neutral red dye (NR) were obtained from Sigma (St Louis, MO, USA). Cytotoxicity detection kit of lactate dehydrogenase enzyme (LDH) was achieved from Roche (Darmstadt, Germany). DMEM-F12 medium, penicillin/streptomycin solutions, fetal bovine serum (FBS), Trypsin -EDTA, were obtained from Invitrogen (Carlsbad, CA, USA). 

*Venom preparation*

The freeze dried venom was dissolved in distilled water and centrifuged at 15000 rpm for 15 min. The supernatant was separated from solution. 

*Cell culture*

Cells were cultured in DMEM/F-12 medium in the presence of penicillin-streptomycin 1% (v/v) and FBS 10% (v/v) inactivated by heat. Cells were maintained in CO_2_ 5% and incubated at 37 ˚C.

*MTT assay*

HUVECs were cultured in DMEM-F12 medium in the presence of FBS 10% plus penicillin-streptomycin 1% and incubated in CO_2 _5% at 37 °C. The cytotoxicity of *V. lebetina* crude venom was evaluated using MTT assay (13). HUVECs were seeded in a 96 well plate at 15000 cells/ well and incubated for 24 h to adhere. After discarding the old medium, the cells were incubated in the medium containing various concentrations 1, 5, 10, 20, 40, 80, 120 µg/mL of crude venom. After 24 h incubation, 20 µL MTT (5 mg/mL) was added to each well and cells were incubated for another 3 h. Finally, the culture medium containing MTT solution was removed and the Formazan crystals were dissolved in 100 µL of dimethyle sulfoxide solvent (DMSO). Absorbance was read with a Synergy HT Microplate Reader (Bio-Tek Instruments, Winooski, VT) at 546 nm. IC_50_ also has been calculated by using GraphPad Prism Software (Version 5.0, San Diego, CA, USA). 

*Neutral red uptake assay*

*V. lebetina* crude venom cytotoxicity was determined using neutral red (NR) assay (14). Briefly, cells were seeded into a 96-well plate at 15000 cells/ well. After 24 h cells were treated to 1, 5, 10, 20, 40, 80, 120 µg/mL crude *Lebetina* venom concentrations for 24 h. Then, the wells medium were replaced with a new one containing NR (40 µg/mL). After 3 h incubation, neutral red medium was removed and the cells were washed with PBS for the remained dye. Finally, neutral red destain solution (50% from ethanol 96%, deionized water 49% and glacial acetic acid 1%) was added to each well and plate was shaking gently for 20 min. Optical density (OD) of neutral red extract was read with a Synergy HT Microplate Reader (Bio-Tek Instruments, Winooki, VT) at 540 nm. 

*LDH release assay*

Cytotoxicity induced by *V. lebetina* crude venom was also assessed by LDH release into the culture medium. The HUVECs were treated at 1, 5, 10, 20, 40, 80, 120 µg/mL crude venom concentrations for 24 h. After incubation, the cells media were transferred into corresponding wells which were optically clear with 96-well flat bottom plate. Released LDH in the media was measured with the Cytotoxicity detection Kit (Roche Diagnostics, Mannheim, Germany). The maximum amount of LDH release was determined by the lysis of cells exposure to 1% Triton X-100 and then was normalized to total LDH content. The absorbance was recorded using a Synergy HT Microplate Reader (Bio-Tek instruments, Winooski, VT) at 490 nm. 

*Morphological studies*

Following overnight incubation of the cells with venom, various morphological alterations and cell damage were qualitatively investigated using a light phase contrast microscope and their photos were taken with a digital camera.

*Data analysis*

Results are expressed as mean ± SD of four replicates. IC_50 _has been calculated by fitting the data to log (inhibitor) vs. response equation. All data analyses were performed using GraphPad Prism version 5.0. 

## Results

*MTT assay*

Anti proliferative and cytotoxicity of *V. lebetina* crude venom were shown by cell count using MTT assay. Data obtained from *in-vitro* analyses showed potential cytotoxic effect on human umbilical vein endothelial cells. In this study, it was determined that *V. lebetina* venom induced a concentration- dependent inhibition of HUVECs (Figure 1-A) with an IC_50_ value of 11.77 μg/mL after 24 hours incubation (Figure 1-B).

*Neutral red uptake assay*

In this colorimetric assay, normal cells could take up and store the neutral red dye in their lysosomes, which can be released in the presence of solublization buffer. Cells with intact lysosomes hold more neutral red dye than dead cells (14). Figure 2 shows an increase in HUVECs growth inhibition in presence of *V. lebetina* crude venom from 1 to 120 μg/mL in a concentration- dependent manner during 24 h incubation. IC_50_ has been calculated 10.52 μg/mL (Figure 2-A, B). 

*LDH release assay*

Cytosolic lactate dehydrogenase (LDH) enzyme released in the media can be measured by dying cells possessing compromised cell membranes. Figure 3 shows LDH release from HUVECs incubated with various concentrations of *V. lebetina* crude venom (1-120 μg/mL) for 24 h. Cells incubated with 20 μg/mL of *V. lebetina *crude venom show 44.4% cytotoxicity. Further, high concentration (120 μg/mL) of the venom caused a significant toxicity (82.8%) during 24 h incubation. 

*Effect of V. lebetina crude venom on the HUVECs*

There were morphological changes between HUVECs exposed to *V. lebetina *crude venom at 10-120 µg/mL concentrations in comparison with control. The microscopic cellular examinations of the HUVECs treated with *V. lebetina* crude venom indicated a rounding shape (Figure 4). These changes led to detachment of the cells from the flask surface and cell monolayer disruption concentration dependently. These morphological alterations increased in high concentration and decrease of total cells number. Finally our results showed *V. lebetina* crude venom induced an inhibition of HUVECs growth with respect to morphological changes.

## Discussion

Snake venoms are a mixture of enzymatic and non-enzymatic agents accompany with highly biological active toxins that can affect synergistically or individually in mammalian cells (15). In this study we evaluated the cytotoxicity of *Vipera Lebetina* crude venom by exposing the HUVECs to various concentrations of crude venom for 24 hour. The results clearly showed that *Vipera Lebetina* venom has significant toxic effects on HUVECs. It was indicated by concentration- dependent inhibition of HUVECs growth in all the tests including MTT, Neutral red and LDH. The cytotoxic effects of *V. lebetina* venom observed in the present study are in accordance with the results obtained by Nalbantsoy *et al*. (2012) (16) that showed the incubation of crude venom *Macrovipera lebetina lebetina* with Mouse fibroblastic (L929) cell-line can inhibits cell proliferation in a concentration- dependent manner. The snake crude venom toxicity recently has also been investigated in other cell lines such as human fibroblast cells (17), renal epithelial (MDCK) cells (18), and endothelial cells (19). Crotalidae and Viperidae are two snake families that their envenomation can cause local as well as systemic bleeding in victim. This is possibly due to direct action of snake venom components such as metalloproteinases, phospholipase A_2_, L- amino acid oxidases activity on the endothelial cells and microvasculature (20, 21, 22 and 23). The pathogenesis of this phenomenon has been investigated extensively. However, the exact hemorrhagic mechanism of action is not clear yet (24). It has already been shown that endothelial cells treated with snake venom can lose cytoplasmic substances lead to cytoplasmic blebs formation (11, 20, and 24). Then endothelial cells become very thin and some lesions will be developed. As a result, cells and other blood components escape from blood circulation to tissues surrounding space (25, 26). Direct effects of hemorrhagic toxins on endothelial cells also showed by Ownby and Geren, (1978) (27) and Gutierez and lomonte, 1989 (28). In our study, crude venom induced toxicity and morphological changes in HUVECs which examined by phase-contrast microscopy (Figure 4). These tests are selected for cytotoxic evaluation of the crude venom. The MTT assay is considered as standard *in-vitro* cytotoxicity (29, 30). According to our results we found a significant toxic activity leading to cell morphological changes with detachment of rounded cell shape. This finding is in favor of distinct effect causes by snake venom fractions as it is described by Rucavado *et al*. (1999) (31). They revealed crude venom and the hemorrhagic toxin (LHF-II) isolated from *Lachesis muta muta *snake venom is not directly related to cytotoxicity of the venom on murine capillary endothelial cells. On the other hand, Borkow *et al.* (1994) (32) was found that the number of venoms studied were not cytotoxic on endothelial cells even during long time of incubation. However, results obtained in our study show *V. lebetina* crude venom can cause significant reduction in endothelial cells viability. To estimate the number of viable cells we used neutral red uptake assay. The basis of this assay is the coupling of viable cells with neutral red dye accumulated into the lysosomes (14). The reduction in cell uptake of neutral red dye combined with the observed morphological changes indicates that the *V. lebetina *crude venom increases cell inhibition in HUVECs. In Figure 3, an obvious increase in cell inhibition during 24 h was accompanied by a more enhance in the leakage of LDH. These results clearly showed significant inhibition of cells up to 80% using MTT and LDH assays. The similarity in inhibition of cell growth by these two methods of assay can be good indicators of cytotoxic effect of crude venom of *V. lebetina* by the mechanism of necrotic effect rather than apoptotic nature of venom (33). As it can be seen in Figure 3, cell inhibition was increased during 24 hours accompany with a greater enhance in LDH leakage. Possibly, venom cellular toxicity can cause cell death and membrane injury which may eventually lead to rupture of the cell membrane and release of LDH. Inflammation and destruction of intracellular organelles are characteristic of cellular events that are called necrotic cell death. This problem may eventually cause rupture of the cell membrane (34). Thus based on the results obtained in the present study, the local and systemic signs of the snake bite by *Vipera Lebetina* including edema, hemorrhage and necrosis may be at least partially associated with the level of vascular toxicity and endothelial cell disruption. Hence the patients bitten by this snake can be at risk of internal bleeding without any previous injury.
